# Fledglings in p53 signaling network

**DOI:** 10.18632/oncotarget.19229

**Published:** 2017-07-14

**Authors:** Takashi Tokino, Masashi Idogawa, Yasushi Sasaki

**Affiliations:** Department of Medical Genome Sciences, Research Institute for Frontier Medicine, Sapporo Medical University, Sapporo, Japan

**Keywords:** p53, target gene, targetome, COL17A1

Since its discovery in 1979, researchers have strived to identify gene targets of the transcription factor p53 [1]. Thus far, more than 200 genes have been identified as p53 targets, supporting the notion that p53 is at the core of the signaling network governing the cell-intrinsic tumor suppression system [2]. However, p53 shows varied topologies of gene regulatory networks in response to a large array of inputs, and it is thought that some missing pieces are still required to complete the ingenious molecular puzzle that underlies the system.

In the current issue of Oncotarget, Matsuda and colleagues systematically analyzed the p53 targetome in two different studies [3, 4]. In the first study, they screened p53-induced genes using microarray data from Adriamycin (ADR)-treated MCF10A breast epithelial cells and RNA sequencing (RNA-seq) data from Breast Invasive Carcinoma (BRCA) in The Cancer Genome Atlas (TCGA) [5]. As a result, 17 genes, including 7 known p53 targets, were highlighted as p53-induced gene candidates. Among 10 novel p53 target gene candidates, they focused on *COL17A1*, whose product, the COL17 protein, is an essential transmembrane component of type I hemidesmosomes and functions as a cell-matrix adhesion molecule [6]. p53 transactivated *COL17A1* through a consensus p53 binding motif in intron 1 of the gene in MCF10A cells after ADR treatment. The p53-dependent induction of *COL17A1* was also observed in the ADR-treated breast cancer cell lines HBL-100 and HBC4, as indicated by the measured mRNA and protein levels.

Why was *COL17A1* recruited onto the p53 team? The authors revealed that exogenous expression of COL17 clearly suppressed cell migration. In addition, ectodomain shedding of COL17 impeded cell invasion. These results seem to strongly indicate that the p53-COL17A1 pathway plays a pivotal role in preventing tumor metastasis. In line with this view, the TCGA-BRCA data showed that *COL17A1* mRNA levels were significantly lower in metastasized breast cancer cells than in corresponding primary breast cancer cells. Taken together, the findings reported by Yodsurang et al. [3] provide new insight into the p53-mediated tumor suppression system and a step forward to complete the p53 puzzle.

In contrast to the p53-induced targets, the list of genes repressed by p53 remains incomplete; p53-repressed genes account for less than 20% of the p53 targetome, as reported thus far [2]. In the second study, Miyamoto et al. [4] undertook the identification of p53-repressed genes using the same data sets (i.e., MCF10A and TCGA-BRCA) supplemented with additional RNA-seq data of mammary glands from *p53*^−/−^ and *p53*^+/+^ mice [7]. This comprehensive meta-analysis identified 44 genes as p53-repressed gene candidates. During p53 activation, p53 change the expression level of various genes, many of which are found in a central gene module of the p53 signaling network that is co-regulated in various cells. Miyamoto et al. [4] revealed that 28 of the 44 genes were simultaneously repressed in a p53-dependent manner in at least two different breast cancer cell lines after ADR treatment, as indicated by the measured mRNA levels. This result suggests that these 28 genes constitute the central gene module of p53-repressed genes in ADR-treated breast cancer cells. Eleven of the 28 genes were first confirmed as p53-repressed genes on an experimental basis. Gene Ontology analysis revealed that cell cycle-associated terms were prominently represented in this set of genes. Notably, the TCGA-BRCA database showed that higher expression of 9 of the 28 genes correlated with worse prognosis in patients with breast cancer, suggesting that strict inhibition of p53-repressed genes can contribute to better survival rates for patients with breast cancer.

How does p53 repress these downstream genes? Based on accumulating evidence, p21/CDKN1A is one of the key molecules of the p53-mediated gene repression system [8]. Consistent with previous studies, Miyamoto et al. [4] found that the p53-mediated gene repression system was impaired in the absence of p21/CDKN1A. However, p21/CDKN1A downregulation via knockdown and knockout techniques did not completely release p53-mediated repression, indicating that other factors may cooperate with p21/CDKN1A. Collectively, these studies have refined our knowledge about the gene network controlled by p53.

The two reports by Matsuda and colleagues undoubtedly extend our understanding of the p53 signaling network. Many transcriptome analyses, including these two studies, have clearly revealed that p53 modulates the expression levels of various genes simultaneously in response to a specific input signal. However, owing to the assiduous efforts of the scientific community to unveil the p53 targetome, we are now being confronted by a fascinating biological question: which set of genes needs to be regulated to execute the appropriate function of p53 under a specific condition? The genes co-regulated by p53 are involved in various cellular functions, including apoptosis, cell cycle arrest, and metabolism. For instance, is the induction of genes related to cell cycle arrest and metabolism necessary to execute apoptosis? If these genes do need to be expressed to allow apoptosis to occur, why has p53 created such an inefficient system? Understanding the gene set that is co-regulated by p53 and its relevant physiological significance under various conditions will open up new avenues of research aimed at elucidating the complex p53 signaling network.
